# Controlling the Host Response in Neurocysticercosis

**DOI:** 10.4269/ajtmh.18-0908

**Published:** 2018-12-31

**Authors:** A. Clinton White

**Affiliations:** Infectious Disease Division, Department of Internal Medicine, University of Texas Medical Branch, Galveston, Texas

Neurocysticercosis is now recognized as a major cause of neurologic disease worldwide. In the United States, reviews of national inpatient data suggest that there are approximately 2,000 patients hospitalized a year with neurocysticercosis, with hospitalization costs of nearly 100 million dollars per year.^1,2^ A similar number of cases are likely managed as outpatients.^3^ These are by far the largest numbers for any parasitic or imported disease in the country. Similarly, data suggest that about 30% of the burden of seizures in endemic areas is due to neurocysticercosis. These endemic areas include most of the world’s population.^4,5^

Over the past two decades, a number of clinical trials have clarified our understanding of optimal management of neurocysticercosis.^6^ A key insight was the recognition that, although always caused by infection of the brain with the larval stage of *Taenia solium*, neurocysticercosis represents a spectrum of disease. The clinical manifestations, optimal treatment, and even pathogenesis vary with the number, location, and viability of the parasites as well as with the host response. Management differs for the different forms of disease, including single enhancing cysts, multiple parenchymal cystic lesions, calcified parenchymal lesions, ventricular cysticerci, and subarachnoid cysticerci. There are important roles in management for antiparasitic drugs, neurosurgery, anti-inflammatory treatments, and anti-epileptic drugs. The balance of these treatments, however, differs with the form of infection. For example, single enhancing lesions are a fairly benign form of neurocysticercosis, but the incidence of recurrent seizures is lessened by treatment with a short course of corticosteroids plus albendazole.^7^ By contrast, ventricular neurocysticercosis is optimally treated with minimally invasive surgery, whereas subarachnoid infection often requires prolonged courses of anti-inflammatory and antiparasitic therapy.

The host inflammatory response plays an important role in the pathogenesis of neurocysticercosis. In fact, most symptoms in neurocysticercosis are caused by the host response. For example, seizures in neurocysticercosis seem to be mediated primarily by the inflammatory response and the neuropeptide substance P.^8^ Similarly, many of the complications of subarachnoid neurocysticercosis, including communicating hydrocephalus, arachnoiditis, and stroke, are mediated by the host inflammatory response.

Despite the prominent role of the host response in neurocysticercosis, there are little high-quality data to guide anti-inflammatory therapy. Randomized trials have demonstrated benefit when corticosteroids are used with albendazole in single enhancing lesions.^7^ Corticosteroids have been used to blunt the inflammatory response after antiparasitic treatment of parenchymal neurocysticercosis.^9^ A single controlled trial suggested that higher doses of dexamethasone might decrease seizures after treatment better than standard doses.^10^ Case series have documented improved survival for subarachnoid neurocysticercosis treated with prolonged courses of antiparasitic drugs and corticosteroids compared with historical controls.^6^ However, doses and durations have varied.

Corticosteroids are associated with a high rate of complications even with short-term use, including altered mental status (steroid psychosis), metabolic bone disease (osteopenia, fractures, and avascular necrosis), sepsis, and hyperglycemia.^11^ Chronic use increases the risk of opportunistic infections. In many inflammatory diseases, other agents have been used as a way of avoiding complications of chronic corticosteroids (as steroid sparing agents). In neurocysticercosis, previous studies have proposed the use of methotrexate as a steroid-sparing agent.^12^ Although there is limited anecdotal experience favoring this approach, some physicians have expressed concerns about the limited efficacy.

In this issue, Nash and and others^13^ now report their experience using the tumor necrosis factor alpha inhibitor etanercept in neurocysticercosis. Most of the studied patients suffered from subarachnoid neurocysticercosis and had developed side effects of corticosteroids or recurrent symptoms when the steroid dose was decreased. The patients seemed to have tolerated the treatment, often with dramatic improvement in symptoms. Most also received methotrexate. Although these data are intriguing, they are anecdotal, with no comparison group. However, these authors have also used etanercept in a porcine model of neurocysticercosis, with mitigation of the inflammatory response to antiparasitic therapy.^14^ Because subarachnoid neurocysticercosis usually requires prolonged antiparasitic and anti-inflammatory therapy, this novel alternative to chronic corticosteroids may represent a significant advance for the field.

The authors also report on using etanercept in a small number of patients who had symptoms associated with calcified cysticerci with perilesional edema. These patients suffered from headaches and/or seizures. In this form of neurocysticercosis, imaging studies demonstrate a calcified granuloma with surrounding edema and contrast enhancement. In a limited number of biopsies, the granulomas contain remnants of the parasites, and edema appears to reflect an acute inflammatory response, perhaps due to an immune response to the release of parasite antigens. The traditional approach to management of these patients would be symptomatic therapy (e.g., anti-epileptic drugs for seizures). Some clinicians have treated patients with symptoms and perilesional edema with anti-inflammatory drugs and noted improvement, but worsening of symptoms often accompanied corticosteroid tapering. There is even evidence that steroid withdrawal may be a factor precipitating episodes of perilesional edema.^15^ In this context, etanercept treatment also was associated with symptomatic improvement. However, the benefit of etanercept over symptomatic therapy requires further study.

A limitation of the present study was that it reports uncontrolled data from patients with a spectrum of illness treated with varied regimens. Despite this limitation, these important observations should modify patient care. The data strongly support the use of etanercept as a steroid-sparing agent in patients with subarachnoid neurocysticercosis. More data are needed to clarify optimal regimens, such as the duration of therapy or the need for coadministration of corticosteroid and/or methotrexate. Cost may also be a consideration for these patients, who often lack private insurance. Etanercept may also play a role in patients with symptomatic calcified lesions with perilesional edema. However, at this point it is unclear whether anti-inflammatory therapy is better than symptomatic therapy alone for these patients. Neurocysticercosis remains a challenge to the clinician, and there is much that we have to learn on optimal management of this complex disease.
